# Editorial: Language development behind the mask

**DOI:** 10.3389/fpsyg.2023.1205215

**Published:** 2023-05-03

**Authors:** Sónia Frota, Núria Esteve-Gibert, Monika Molnar, Marina Vigário

**Affiliations:** ^1^Center of Linguistics, School of Arts and Humanities, University of Lisbon, Lisbon, Portugal; ^2^Faculty of Psychology and Education Sciences, Open University of Catalonia, Barcelona, Catalonia, Spain; ^3^Speech-Language Pathology, University of Toronto, Toronto, ON, Canada

**Keywords:** face masks, COVID-19, language development, speech processing, auditory speech cues, audio visual integration, speech production, speech perception

Language includes both auditory and visual cues relevant to language learning. Human communication and interaction rely on the acoustic speech stream produced as well as on language related visual information, most prominently the hands and the mouth and eye regions in the face. Infants and toddlers have been shown to integrate different sensory perceptual cues, and take advantage of these cues very early in development (e.g., Lewkowicz and Hansen-Tift, 2012; Choi et al., 2018; Pons et al., 2019). The COVID-19 pandemic has affected human communication through the pervasive use of masks. Masks degrade the quality of the speech signal (e.g., Bottalico et al., 2020; Rahne et al., 2021; Thibodeau et al., 2021), while also rendering facial cues to language inaccessible, particularly those pertaining to the mouth region (Singh et al., 2021). Therefore, since the beginnings of 2020 language learners have been exposed to sets of auditory and/or visual cues to language that differ from those commonly available in the ambient language. Face masked interaction and communication may also impact other aspects of communicative behavior with consequences to child cognitive development (Deoni et al., 2022). Developmental research faces the challenge to understand whether and when these potential effects take place in development, across different populations.

The COVID-19 pandemic also offered an unprecedented opportunity to study how the quality of auditory and visual cues, as well as their interplay and integration, shape language development and human communication. It is known that changes in selective audiovisual attention are linked to language development, language (un)familiarity, speaker characteristics, or increased processing effort (e.g., Morin-Lessard et al., 2019; Pejovic et al., 2019). For adults, masking is challenging for signers, the hearing impaired, as well as the normal hearing, and its consequences are modulated by speaking style (Chodosh et al., 2020; Cohn et al., 2021).

This Research Topic promoted innovative research on the effects of face masked speech on language development and communication. The seven papers included in the Research Topic analyze the effects of different types of face masks on speech processing, both on the auditory-only and audiovisual modalities, considering various communication settings and populations, including infants, children with normal hearing, children with hearing loss, and adults. The research papers are authored by 35 contributors from a multidisciplinary background (linguistics, psycholinguistics, psychology, speech perception, auditory research, cognitive science, clinical medicine, education).

Pycha et al. examined how a fabric face mask affected speech intelligibility according to speaking style, background noise, and visual information indicating whether the speaker was wearing or not a face mask. Their findings that adult speakers and listeners adjust their speech production and comprehension to fit the demands of the communicative context have implications to theories of speech production and perception, and raise interesting questions for face-masked speech and language learning.

The question of whether and how face masks might disrupt communication, especially in educational settings, was addressed in four research papers, targeting different populations and age ranges. Lalonde et al. investigated the effects of a surgical mask, fabric mask, ClearMask^TM^ and The Communicator^TM^ on auditory-only and audiovisual speech in adults with normal hearing, children with normal hearing and children with bilateral hearing loss (aged between 7 and 18 years). Their results from a consonant identification task were similar across groups, showing that face masks negatively impact speech understanding in children, and surgical masks are probably the least impacting in a classroom setting where children do not always orient to the speaker. Schwarz et al. addressed the extent to which the acoustic and visual effects of a fabric mask could be mitigated by semantic predictability in children (aged 8–12) and adults. Although face-masked speech led to more mistakes in a sentence-final word repetition task, high semantic predictability fully compensated for the effects of face mask in adults and partially in children. The authors conclude that the availability of contextual information might help overcome negative effects of face masks in classroom settings. Mitsven et al. studied the effects of face masks on the speech heard and produced in the classroom by 3.5–4.5-year-old children with and without hearing loss. The speech of teachers was more affected than children's language production, showing compensatory strategies whereby teachers produced more words per minute during COVID-19, and children with hearing loss were overall exposed to longer and more diverse speech. Finally, in a cross-cultural study, Crimon et al. asked whether mask wearing in nursery schools impacted how educators interacted with children under 3 years of age. They reported that educators perceived changes in their communication behavior, in the quantity (decrease) and quality (increase) of speech produced, as well as in the (increased) use of non-verbal cues. The potential effects of such changes on language development are unknown.

The two remaining papers in this Research Topic examined speech processing in infants. Orena et al. investigated infants voice-face recognition abilities in the presence of unfamiliar voices when the speaker's face is partially occluded. Unlike at 24 months, at 12 months infants were not able to recognize an unfamiliar voice when visual access to the speaker's mouth is blocked. Frota et al. addressed the impact of face-masked speech and other COVID-related changes in the early word segmentation abilities of infants born during COVID-19. They found no segmentation in 7–9-month-olds in either an auditory or audiovisual task with and without an FFP2 mask, contra pre-pandemic findings, together with lower scores on measures of language and cognitive development compared to pre-COVID data.

Overall, the papers in this Research Topic suggest adverse face mask effects on communication and language learning, that might be overcome with compensatory strategies, especially in the case of adults and older children. However, the impact of face masked interaction and communication on younger children seems more stringent, and future research needs to examine its actual effects on later language outcomes. We hope that this Research Topic will pave the way to further (longitudinal) research on how COVID-19 might have potentially affected language development, and how educators, practitioners and families may help overcome such effects.

## Author contributions

SF prepared the first draft of the manuscript. All authors contributed to the article and approved the submitted version.
